# The impact of mindfulness training on infection prevention practices in intensive care units

**DOI:** 10.1017/ash.2025.174

**Published:** 2025-05-19

**Authors:** Anucha Apisarnthanarak, Panuch Eiamprapaporn, Punchong Hanvivadhanakul, Watcharee Chancharoenrat, Suphannee PhetSaen, Tipanan Tamsuan, Kittiya Jantarathaneewat, Piyaporn Apisarnthanarak, Linda M. Mundy, Aunchaya Yeamsukon, Duangrutai Aksarachaipruk, Isaree Naisangunsri, Ven. Surapoj Saddhadhigo Bhikkhu, Ven. Tawatchai Dhammadipo Bhikkhu

**Affiliations:** 1 Division of Infectious Diseases, Faculty of Medicine, Thammasat University Hospital, Pratum Thani, Thailand; 2 Department of Medicine, Faculty of Medicine, Thammasat University Hospital, Pratum Thani, Thailand; 3 Department of Infection Control, Thammasat University Hospital, Pratum Thani, Thailand; 4 Department of Radiology, Siriraj Hospital, Bangkok, Thailand; 5 Mount Sinai Health and NYC Health+Hospitals, NYC, NY, USA; 6 The Mind Development in Honor of His Majesty the King, Baan Wang Muang, Phang-Nga province, to dedicate merit to Their Majesties, the King and the Queen, Thailand

## Introduction

The practice of mindfulness is described as paying attention to the present moment.^1^ Mindful awareness is meant to help transition from a state where thoughts and actions are in automatic mode to a state where situations are observed, acknowledged, and accepted.^2^ A recent systematic review identified that brief mindfulness interventions aimed at healthcare workers (HCWs) improved a myriad of clinical outcomes.^3^ Mindfulness practices may help HCWs experience defined as “brief periods of time in which people experience a deep interconnectedness” linked with care of with patients.^4,5^

Limited data are available on the impact of mindfulness training on infection prevention (IP) practices. The role modeling of mindful hand hygiene within a clinical setting may have a cascading effect on improving peers adherence to IP practices.^6^ Further, adoption of mindful practice, may have other benefits including enhanced attention, situational awareness, provider well-being, and reduction of medical errors by addressing cognitive biases.^7,8^ We report the design, execution, and analysis of a quasi-experimental study that evaluated the association of mindfulness training on change in IP practices in 4 intensive care units (ICUs) and the self-reported changes in wellness by participants.

## Methods

A quasi-experimental study was conducted to evaluate the impact of mindfulness training in 2 surgical and 2 medical ICUs at Thammasat University Hospital (TUH); all HCWs consented to participation. The study periods consisted of the six-month pre-implementation period (Period 1) from 12/1/2023–5/31/2023 and the six-month post-implementation period (Period 2) from 6/01/2024–11/30/2024. Mindfulness training was conducted by a multidisciplinary team of study investigators and leadership representatives from The Mind Development in Honor of His Majesty the King.

During Period 1, baseline data collection included age, gender, type of unit, occupation, religion, previous mindfulness training, level of faith in religion, stress, anxiety, feeling light and relieved, peace in daily life, and happiness in daily life. The study outcomes included IP practices, report of medical errors, and HCWs self-reported wellness measures. The IP outcomes were compliance with the observed 5-moments hand hygiene (5MHH), compliance to personal protective equipment (PPE) use as a composite score (Appendix 1), and medical errors defined as total errors reported to hospital administration per month (e.g., forgot to discontinue medication, discontinue the wrong medication). The 5MHH observations were performed by Infection Control nurses per the World Health Organization recommendations,^9^ with at least 50 5MHH observations required monthly for each unit. The five self-reported wellness metrics were level of stress, anxiety, feeling light and relieved, peace in daily life, and happiness in daily life. These wellness metrics were assessed on a 5-point Likert scale and dichotomized with responses of 4 (agree) or 5 (strongly agree) coded as 1 and 0 otherwise and collected at the start of Period 1 and end of Period 2.

During Period 2, participants registered on-line before attending each mindfulness practice session and the sessions began with a 5-minute introduction on how to apply mindfulness in the ICU settings. The trice-weekly 12-minute mindfulness trainings were guided by A.A., P.E., P.H. using 2 video clips developed by The Mind Development in Honor of His Majesty the King (https://www.youtube.com/watch?v=AbAfdJHgKss; https://www.youtube.com/watch?v=DVZTBk6mG84) followed by discussion on mindfulness practice and IP, if questions arise. All mindfulness teachers had a sustained mindful awareness practice of at least three years after initial training with The Mind Development in Honor of His Majesty the King; educational content included mindfulness concepts and integration of the mindfulness training into clinical IP practices to improve 5MHH. Enrolled HCW participants were required to attend at least 1 educational session per week to meet inclusion criteria for the final data analysis. The study was approved by the TUH Institution Review Board.

All analyses were performed using SPSS, version 26 (Armonk, NY). *χ* ^2^tests were used to compare categorical variables. Independent t-tests were used for continuous data. All *P* values were 2-tailed, and *P* < 0.05 was considered statistically significant. A multivariate analysis was conducted to evaluate factors associated with HCWs self-report of wellness for each study period. Adjusted odd ratios (aORs) and 95% confidence intervals (CIs) were calculated.

## Results

Overall, 284 (86.3%) of 329 eligible HCWs in 4 ICUs consented to study participation; 272 HCWs (95.8%) completed full training and met the inclusion criteria for data analysis. The study cohort’s mean age (S.D.) was 31.2 (SD, 8.9) years; the most common self-reported religion was Buddhism and half of participants reported strong faith in their religion. Demographic and baseline characteristics of the study populations are summarized in Table 1.

**Table 1.** Baseline characteristics of 272 intensive care unit healthcare workers who participated in mindful awareness training

Variable	No (%) (N = 272)
Age (years; mean ± S.D.)	30.9 ± 8.7
Gender (female)	236 (87)
**Religion**	
Buddhism	255 (93.8)
Christian	5 (2)
Islamic	9 (3.3)
None	3 (1.1)
**Working experience (year)**	
<5 years	160 (58.8)
≥5 years	112 (41.2)
**Occupation**	
Nurses	223 (82)
Nurse’s assistant	48 (17.7)
Others^a^	1 (0.4)
Strong faith in religion	146 (53.7)

a Housekeeper and ward clerk.

During Period 2, there were significant improvements in the observed IP outcomes and reduction of medical errors (Table 2). There was an increase in the 1^st^ and 4^th^ moments of the 5MHH, improvement in the composite score for full PPE compliance (14.1 (SD, 0.3) vs 13.7 (SD,0.6) points; *P* = 0.01) and reduction in medical errors (7.9% vs 10.9%; *P* = 0.04) (Table 2). For the self-reported wellness outcomes, there was a significant reduction in stress (79.8% vs. 67.3%; *P* = 0.001), reduction in anxiety (76.8% vs. 65.1%; *P* = 0.003), increased feeling of light and relief (47.1% vs. 58.5%; *P* = 0.01), increased report of peace in daily life (52.2% vs 62.1%; *P* = 0.02), and increased report of happiness in daily life (51.5% vs. 61.8%; *P* = 0.01) (Table 2). In the multivariable analysis, work experience ≥5 years (aOR = 3.21; 1.53–6.71) and strong faith in religion (aOR = 2.64; 95% CI = 1.21–5.76) were associated with improvement in all five self-reported outcomes during Period 2.

**Table 2.** Comparison of infection prevention practices, medical errors, and wellness outcomes among 272 intensive care unit healthcare workers during the pre- and post-intervention periods

Outcomes	Pre-intervention	Post-intervention	*P* value
Infection control observations			
**Overall hand hygiene rate (n, %)^a^**			
All^b^	1116/1,436 (77.7)	1361/1,629 (83.5)	0.001
Moment 1	137/247 (55.5)	170/243 (70)	0.001
Moment 2	258/356 (72.5)	351/458 (76.6)	0.19
Moment 3	201/222 (90.5)	194/215 (90.2)	1.00
Moment 4	351/390 (90)	461/492 (93.7)	0.04
Moment 5	169/221 (76.5)	185/221 (83.7)	0.07
**Hand hygiene rate in Medical ICUs (n, %)** ^a^			
All^c^	599/728 (82.3)	772/873 (88.4)	0.001
Moment 1	56/109 (51.4)	65/97 (67)	0.02
Moment 2	159/190 (83.7)	223/253 (88.1)	0.21
Moment 3	81/92 (88)	73/84 (86.9)	0.82
Moment 4	202/223 (90.6)	254/263 (96.6)	0.008
Moment 5	101/114 (88.6)	157/176 (89.2)	0.85
**Hand hygiene rate in Surgical ICUs (n, %)** ^a^			
All^d^	517/708 (73)	589/756 (77.9)	0.030
Moment 1	81/138 (58.7)	105/146 (71.9)	0.024
Moment 2	99/166 (59.6)	128/205 (62.4)	0.594
Moment 3	120/130 (92.3)	121/131 (92.4)	1.000
Moment 4	149/167 (89.2)	207/229 (90.4)	0.737
Moment 5	68/107 (63.5)	28/45 (62.2)	1.000
**Composite score of full PPE compliance (Means ± S.D.)^e^**	13.7 ± 0.6	14.1 ± 0.3	0.013
Medical ICUs	13.6 ± 0.7	14.2 ± 0.3	0.011
Surgical ICUs	13.8 ± 0.5	13.9 ± 0.3	0.489
**Incidental reported of medical error per total reported incidents^f^ (n, %)**	93/851 (10.9)	62/787 (7.9)	0.042
**HCWs Wellness outcomes**			
Stress at work (n, %)	**217/272 (79.8)**	**183/272 (67.3)**	**0.001**
Anxiety at work (n, %)	**209/272 (76.8)**	**177/272 (65.1)**	**0.003**
Feel light and relief (n, %)	**128/272 (47.1)**	**159/272 (58.5)**	**0.01**
Peaceful in daily life (n, %)	**142/272 (52.2)**	**169/272 (62.1)**	**0.02**
Happiness in daily life (n, %)	**140/272 (51.5)**	**168/272 (61.8)**	**0.01**

Note: S.D.; standard deviation, ICU; Intensive Care Unit; HCWs = Healthcare workers;

a Hand hygiene performed per total hand hygiene opportunities observed; at least 50 opportunities of observations of overall 5 moments hand hygiene per unit per month are required. The most common type of hand hygiene in ICUs in this hospital using alcohol hand rub.

b OR (0.69 [95% CI, 0.57-0.83], Power = 99.4%), effect size = 0.2.

c OR (0.61 [95% CI, 0.45-0.81], Power = 93.3%), effect size = 0.2.

d OR (0.77 [95% CI, 0.60-0.98], Power = 58.7%), effect size = 0.1.

e Power 98%, effect size 1.2.

f Per total reports, per month.

## Discussion

The major finding of this study was the association of mindful awareness training with improvement in three of the 5MHH, use of PPE, reduction in reported medical errors, and an increase in five self-reported wellness measures in a cohort of predominantly ICU nurses. The mindfulness training emphasized being fully aware in the present moment and the measured changes occurred over a six-month interval. Notably, these study findings are consistent with and contribute to the evidence reported in prior ICU HCW studies.^7,8,10^ Furthermore, our study findings suggest potential differential growth in mindful awareness for HCWs with longer (>5 years) working experiences and self-reported strong faith, adding to an earlier report of improved wellness in HCWs who acknowledged a strong religious faith.^11^

Study limitations include the inherent biases associated with quasi-experimental studies for potential Hawthorne effect and temporal changes over the 12-month period. Second, generalizability may be limited given that the study is from a single study site, targeted primarily ICU nurses, and the majority of participants (90%) reported Buddhism as their religious faith. Lastly, the nature of self-report outcomes (e.g., wellness matrix) may subject to reporting biases.

In conclusion, the mindful awareness training was associated with observed increases in IP practices, reduction in medical errors, and higher measures of HCWs’ wellness. The intervention was feasible to implement and to integrate into routine ICU healthcare delivery. Future multi-center randomized-control studies on mindful awareness education and training in different settings will inform on sustained HCW healthy IP behaviors and practices.

## Supporting information

Apisarnthanarak et al. supplementary material Apisarnthanarak et al. supplementary material

## Supplementary material

To view supplementary material for this article, please visit https://doi.org/10.1017/ash.2025.174

## References

[1] Kabat-Zinn J. Full catastrophe living: Using the wisdom of your body and mind to face stress, pain, and illness. New York: Bantam Books; 2013:1–9.

[2] Weick KE , Sutcliffe KM , Obstfeld D. Organizing for high reliability: processes of collective mindfulness In: Boin A , ed. Crisis Management. Los Angeles: Sage; 2008:31–66.

[3] Gilmartin H , Goyal A , Hamati M , et al. Brief mindfulness practices for healthcare providers: a systematic literature review. Am J Med 2017;130:1219e1–19e17 10.1016/j.amjmed.2017.05.041 28687263

[4] Pargament KI , Lomax JW , McGee JS , Fang Q. Sacred moments in psychotherapy from the perspectives of mental health providers and clients: prevalence, predictors, and consequences. Spirituality in Clinical Practice 2014;1:248–262

[5] Quinn M , Fowler KE , Harrod M , et al. Exploring sacred moments in hospitalized patients: an exploratory qualitative study. J Gen Intern Med 2023;38:2038–2044 36650333 10.1007/s11606-022-07999-z PMC9845021

[6] Gilmartin HM. Use hand cleaning to prompt mindfulness in clinic. BMJ 2016;352:i13 26728590 10.1136/bmj.i13

[7] Brown KW , Ryan RM. The benefits of being present: Mindfulness and its role in psychological well-being. Journal of Personality and Social Psychology 2003;84:822 12703651 10.1037/0022-3514.84.4.822

[8] Sibinga EMS , Wu AW. Clinician mindfulness and patient safety. JAMA 2010;304:2532–2533 21139116 10.1001/jama.2010.1817

[9] Sax H , Allegranzi B , Chraïti MN , Boyce J , Larson E , Pittet D. The World Health Organization hand hygiene observation method. Am J Infect Control 2009;37:827–834 20004812 10.1016/j.ajic.2009.07.003

[10] Gilmartin H , Saint S , Rogers M , Winter S , Snyder A , Quinn M , Chopra V. Pilot randomised controlled trial to improve hand hygiene through mindful moments. BMJ Qal Saf 2018;27:799–806 10.1136/bmjqs-2017-007359 PMC6701174 29463769

[11] Apisarnthanarak A , Greene MT , Collier KM , Kasatpibal N , Fowler KE , Saint S. The influence of spirituality, religiosity, and self-care on well-being among Thai infection preventionists during the COVID-19 pandemic. Antimicrob Steward Healthc Epidemiol 2024;4:e26 38415096 10.1017/ash.2023.510 PMC10897717

