# Supplementary material for: The impact of mindfulness training on infection prevention practices in intensive care units
Source: Antimicrob Steward Healthc Epidemiol. 2025 May 19;5(1):e116. doi: 10.1017/ash.2025.174 (PMC12089734; doi:10.1017/ash.2025.174)
Supplement: Apisarnthanarak et al. supplementary material [file S2732494X25001743sup001.docx]

**Appendix 1: Composite Score for Full Personal Protective Equipment Compliance**

| **Variable** | **Score** |
| --- | --- |
| Signage for isolation in front of the room and in chart  Available, accessible equipment to use for the patient in isolation  Does not bring patient chart or any documents into the patient room  Check the adequacy of the isolation gown, gloves, mask, alcohol gel  Perform hand hygiene according to 5MHH  Doff gloves after leaving the patient room and washing hand  Doff mask after leaving the patient room and put it in the designated disposal  Limit necessary HCWs to enter the patient room  Limit number of visitors to enter the patient room  Check the appropriateness of waste containers  Changing linin per hospital policy per hospital policy  Perform environmental cleaning per hospital policy  When transfer to OT, notify OT staff  When transfer, notify stretch shift staff  Chief nurse supervision that HCWs comply with the policy list above  **Total score** | 1  1  1  1  1  1  1  1  1  1  1  1  1  1  1  15 |

5MHH = 5-moments hand hygiene; HCWs = Healthcare workers; OT = Operating theatre
